# Section on Psychology

**Published:** 1866-07

**Authors:** 


### SECTION ON PSYCHOLOGY.

*May 2d.*

The members of the Section on Psychology unite in the recommendation that the subject of insanity be again referred to a committee, for a report at the next meeting of this body, and that Dr. Isaac Ray, of the Butler Hospital for the insane, at Providence, R.I., be that committee, if the rules of the Association will allow of a committee of *one*, or, if the committee must be larger, that he be the chairman thereof.

Clement A. Walker, Chairman.

*Committee on Insanity.*—Drs. Isaac Ray, Rhode Island; Wilson Lockhart, Indiana; W. P. Jones, Tennessee; W. H. Stokes, Maryland; A. B. Cabaniss, Mississippi.

